# Gli-1/PI3K/AKT/NF-kB pathway mediates resistance to radiation and is a target for reversion of responses in refractory acute myeloid leukemia cells

**DOI:** 10.18632/oncotarget.8844

**Published:** 2016-04-20

**Authors:** Xiaodong Li, Fang Chen, Qiuhua Zhu, Bingjie Ding, Qingxiu Zhong, Kaikai Huang, Xuejie Jiang, Zhixiang Wang, Changxin Yin, Yufeng Zhu, Zhen Li, Fanyi Meng

**Affiliations:** ^1^ Hematology Department, Nanfang Hospital, Southern Medical University, Guangzhou 510515, Guangdong, China; ^2^ Hematology Department, Kanghua Hospital, Dongguan 523080, Guangdong, China

**Keywords:** radioresistance, refractory AML, LDE225, Gli-1/PI3K/AKT/NF-kB

## Abstract

Total body irradiation combined with chemotherapy is currently the most effective procedure as a preparative myeloablative regimen. However, resistance to radiotherapy and chemotherapy in refractory acute myeloid leukemia is associated with short-time recurrence after allogeneic hematopoietic stem cell transplantation. To address this issue, we used three cell lines, HL60, HL60/ADR (adriamycin-resistant cells), and HL60/RX (a radiation-resistant cell line established from HL60 cells), as cellular models to investigate the mechanism of the Hedgehog (Hh) signaling pathway resulting in radioresistance, and the efficacy of LDE225 (an inhibitor of the Hh pathway) to enhance radiation sensitivity. Our results indicated that HL60/RX and HL60/ADR cells showed an increased in radioresistance and elevated activity of Hh pathway proteins compared with HL60 cells (P<0.001). In addition, LDE225 significantly reduced clonogenic survival with a sensitivity enhancement ratio (SER) of 1.283 for HL60/ADR and 1.245 for HL60/RX cells. The combination of LDE225 with irradiation significantly increased radiation-induced apoptosis and expression of γ-H2AX and BAK compared with single-treatment groups in both HL60/RX and HL60/ADR cells (P<0.001). In vivo, the combination of LDE225 with irradiation exerted a significant antitumor effect compared with the control and single agents in HL60/RX- and HL60/ADR-xenografted mouse models (P<0.001). Furthermore, our data obtained from western blot and IHC analyses showed that the activation of pAKT and NF-kB was reduced by LDE225 treatment in both HL60/ADR and HL60/RX cells. This demonstrates that the Gli-1/PI3K/AKT/NF-kB pathway plays a key role in resistance to radiation, and that inhibition of the Hh pathway sensitizes cells to radiation by overcoming radioresistance.

## INTRODUCTION

Acute myeloid leukemia (AML) is one of the most prevalent cancers with a short survival period, and stem cell transplantation (SCT) continues to be an effective treatment [1, 2]. Total body irradiation (TBI) combined with chemotherapy is currently the most common procedure as a preparative myeloablative regimen [3]. However, there remains a high failure rate in patients who receive TBI before SCT [4, 5]. One of the causes of this treatment failure is remaining radioresistant leukemia cell clones. Therefore, understanding the mechanisms of resistance to radiotherapy and increasing the therapeutic efficacy are significant to devise novel therapies for AML, such as using targeted drugs as radiation sensitizers.

The hedgehog (Hh) signaling pathway plays a key role in embryonic development [6, 7] and is necessary to support tumorigenesis, proliferation, and metastasis of numerous tumor types [8-10]. Many studies have demonstrated that overexpression of Hh signaling genes is linked to radiation resistance, and downregulation can enhance radiation responses in many tumor types including pancreatic, anaplastic thyroid, esophageal, and non-small cell lung cancers [11-14]. In addition, several clinical studies have shown a positive correlation between overexpression of Hh signaling genes and poorer outcomes of various kinds of cancer [15-17]. Because many genes involved in controlling the cell cycle, signal transduction, apoptosis, and repair of DNA damage are regulated by Hh signaling [18], Hh inhibitors are considered to be potential agents to improve radiation responses. For example, targeted inhibition of Hh as an induction treatment followed by irradiation has been reported as a new therapeutic strategy and promising treatment option for basal cell carcinoma [19, 20].

In particular, a recent study has indicated that aberrant Hh pathway signaling is a negative prognostic factor for AML [21]. Other studies have shown that Hh signaling is essential for the survival and drug resistance of leukemia cells [22, 23]. Interestingly, a recent report has demonstrated that inhibition of the Hh pathway with LDE225 sensitizes AML cells to 5-azacytidine, and a clinical trial based on these results is ongoing [24]. However, there are no reports of the effects and mechanisms of Hh pathway signaling on radiation resistance or the application of inhibitors to AML. In the present study, we hypothesized that disruption of Hh signaling could increase the sensitivity of radiation-resistant leukemia cells to ionizing radiation. The results demonstrated an association between overexpression of Hh signaling and radiation resistance, and Hh inhibition can enhance radiosensitivity. Therefore, the Hh pathway is an efficient target to enhance responses to radiation in AML.

## RESULTS

### Expression of the Hh signaling pathway and radiosensitivity of HL-60, HL-60/RX, and HL-60/ADR cells

**Figure 1:**
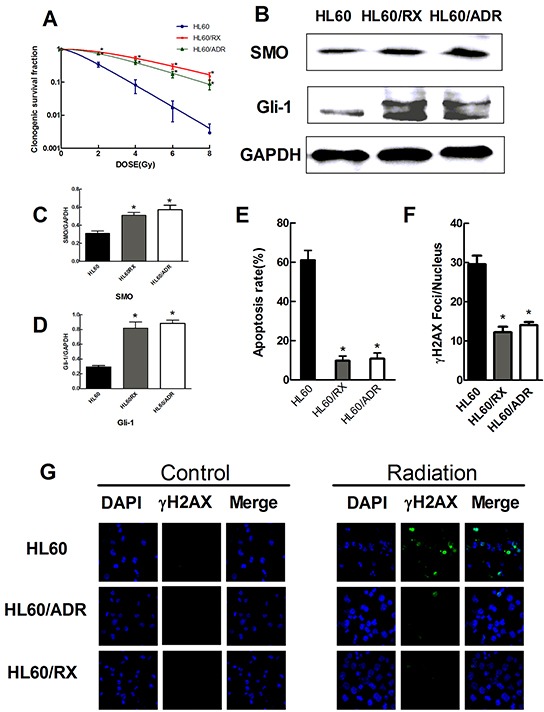
Radiation-resistant HL60/RX cells and adriamycin-resistant HL60/ADR cells show an increased radioresistance and increased activity of the Hedgehog pathway than HL60 cells **A.** Survival curves of HL60, HL60/ADR, and HL60/RX cells after radiation. HL60/ADR and HL60/RX cells show great resistance to radiation than the parental cells. **B–D.** The protein expression of SMO and Gli-1 in HL60, HL60/ADR, and HL60/RX cells. GAPDH was used as loading control. **E.** The percentage of apoptosis cells in HL60, HL60/ADR, and HL60/RX cells after radiation (4.8Gy). **F.** Average number of γ-H2AX foci/nucleus after radiation (4.8Gy). **G.** γ-H2AX formed in the nucleus was more significant in HL60 cells compared with HL60/ADR and HL60/RX cells. *, p<0.001 vs HL60.

To investigate the role of the Hh signaling pathway in radiation resistance of leukemia cells, we established a radiation-resistant cell line (HL-60/RX) from HL-60 cells (Table 1). First, we detected the expression of Smoothened (SMO), a key transducer of the Hh signaling pathway, and Hh target protein Glioma-associated oncogene family zinc finger 1 (Gli-1) in all three cell lines. Then, clonogenic assays were performed to investigate their responses to radiation. The surviving fraction (SF) was calculated as follows: SF=colonies counted/(cells seeded×plating efficiency). The survival curves of cell lines after irradiation are illustrated in Figure 1A. The dose quasithreshold (Dq) and mean lethal dose (D0) values were 1.134±0.456 Gy and 1.282±0.271Gy for HL60 cells, 4.513±0.804 Gy and 3.033±0.29 Gy for HL-60/RX cells, and 3.310±0.677 Gy and 2.437±0.259 Gy for HL-60/ADR cells, respectively (Table 2). Additionally, the expression of SMO (Figure 1C) and Gli-1(Figure 1D) in HL60/RX cells was significantly higher than that observed in HL60 cells (P<0.001) and similar to that in HL60/ADR cells. These results demonstrated that HL-60/RX and HL60/ADR cells have remarkable resistance to radiation compared with HL/60 cells (P<0.001), and suggest that activation of the Hh signaling pathway may be associated with the resistant phenotype.

**Table 1:** Dose selection for establishment of HL60/RX cells (Gy)

Iteration	1	2	3	4	5	6	7	8	9	10	11	12
Radiation	2.5	2.5	3	3	3.3	3.3	4.2	4.2	4.8	4.8	4.8	4.8

**Table 2:** Radiation survival curve parameters for HL60, HL60/RX, and HL60/ADR cell lines (mean ± SD)

Cell lines	D0(Gy)	Dq(Gy)
HL60	1.282±0.271	1.134±0.456
HL60/RX	3.033±0.290*	4.513±0.804*
HL60/ADR	2.437±0.259*	3.31±0.677*
P value	<0.001	<0.001

* P<0.001 vs. HL60.

### γ-H2AX foci and the apoptosis rate after irradiation of HL-60, HL-60/RX, and HL-60/ADR cells

To further explore the mechanism of radiation resistance, we measured the apoptosis response to a single dose of 4.8 Gy in HL60/RX, HL60/ADR, and HL60 cells (the parent cell line). At 48 h after irradiation, the apoptosis ratio was significantly higher in HL60 cells (61.0%) than in HL60/RX (9.7%) and HL60/ADR (10.8%) cells (Figure 1E). These data support that the enhanced radiation resistance in radioresistant sublines is linked to a decrease in apoptosis.

γ-H2AX is one of the earliest markers in response to a DNA double-strand break (DSB), and dephosphorylation of γ-H2AX occurs after DNA repair [25]. Therefore, γ-H2AX foci are often recognized as a DNA damage marker after irradiation. The expression of γ-H2AX in all three cell lines is shown in Figure 1G. The γ-H2AX-positive level (Figure 1F) in HL60 cells exhibited a significant increase compared with that in HL60/RX and HL60/ADR cells at 24 h after exposure to radiation (P<0.001). These findings provide support that HL60/RX and HL60/ADR cells have an increased ability to repair DNA damage compared with the parent line.

### LDE225 significantly enhances the radiosensitivity of HL60/RX and HL60/ADR cells

**Figure 2:**
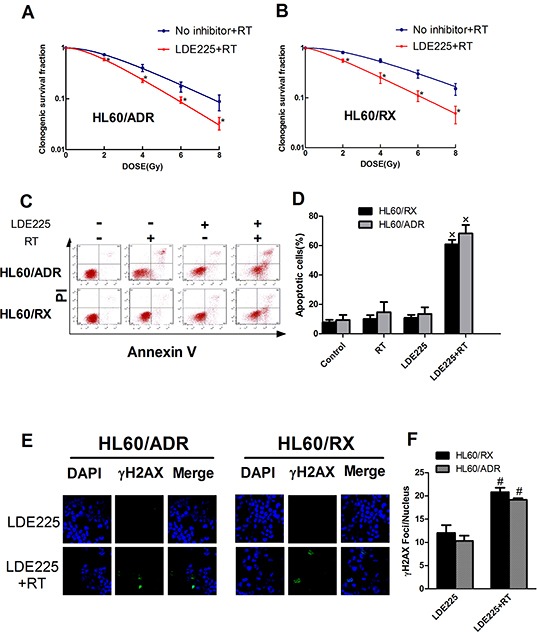
Inhibition of Hedgehog pathway enhances the radiosensitivity both in radio-resistant leukemia and drug-resistant leukemia cells **A, B.** Survival curves of HL60/ADR and HL60/RX cells after radiation. **C, D.** Cell apoptosis distribution showed that the percentage of apoptosis cells significantly increase in cells treated with LDE225 and radiation combined with control, RT alone and LDE225 alone. **E, F.** The highest increased in γ-H2AX expression was seen in HL60/ADR and HL60/RX cells treated with LDE225 and radiation.*, p<0.001 vs No inhibitor+RT. ×, p<0.001 vs vehicle-treated control group, radiation group, or LDE225 group. #, p<0.01 vs LDE225 group. Abbreviation: RT, radiation.

To examine the effect of the Hh pathway on radiation resistance, HL60/ADR and HL60/RX cells were pretreated with LDE225 (a novel synthetic Smo antagonist) or DMSO for 48 h, followed by irradiation. The results showed a significant decrease in the clonogenic SF at different radiation doses in both HL60/ADR and HL60/RX cells after exposure to LDE225 (Figure 2A, 2B). The SERs were 1.283 for HL60/ADR cells and 1.245 for HL60/RX cells. Furthermore, as shown in Figure 2C and 2D, simultaneous treatment with LDE225 and radiation significantly increased the percentage of apoptotic cells in comparison with radiation alone, LDE225 alone, and the control. Additionally, the number of γ-H2AX foci in cells treated with LDE225 and radiation was significantly higher than that in cells treated with LDE225 alone Figure 2F. These results indicate that the Hh pathway plays an important role in radiation resistance, and that an inhibitor promotes a significant radiation response in radiation-resistant leukemia cell lines.

### LDE225 pretreatment sensitizes HL60/RX and HL60/ADR cells to radiation via inhibition of the PI3K/AKT/nuclear factor kappaB (NF-kB) pathway

**Figure 3:**
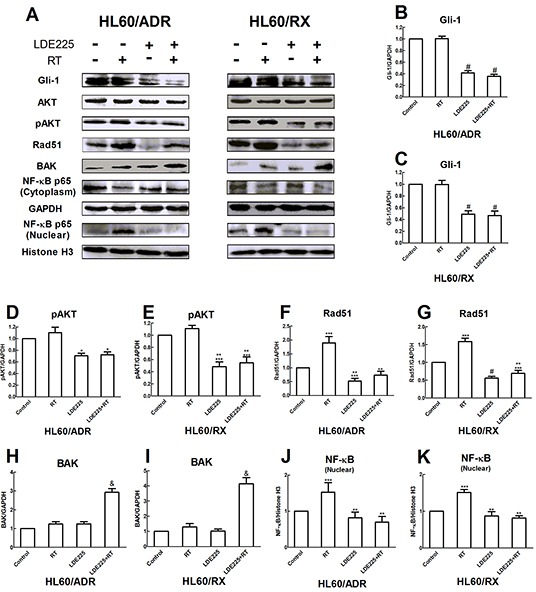
LDE225 enhances the radiosensitivity by downregulating the activation of pAKT and NF-kB in HL60/ADR and HL60/RX cells **A.** Cells were treated with vehicle alone, radiation alone, LDE225 alone, and combination therapy (LDE225+radiation) respectively. The protein expression of Gli-1, AKT, pAKT, NF-kB, Rad51, BAK were analyzed by western blot. The quality of separation was monitored using GAPDH as mark **B–I.** and Histone H3 **J, K.** Each bar represents the mean ± SD. #, p<0.001 vs vehicle-treated control group or radiation group; *, p<0.01 vs vehicle-treated control group or radiation group **, p<0.001 vs radiation group; ***, p<0.01 vs vehicle-treated control group; &, p<0.001 vs vehicle-treated control group, radiation group, or LDE225 group. Abbreviation: RT, radiation.

To further explore the mechanism of LDE225 in enhancing radiosensitivity of HL60/RX and HL60/ADR cells, we measured the expression of phosphorylated AKT (pAKT), NF-kB, Rad51, and BCL2-antagonist/killer (BAK) in the cells by western blot analysis after treatment (Figure 3A). As shown in Figure 3B and 3C, after 48 h of pretreatment with LDE225, a significant decrease in Gli-1 expression was observed in both HL60/ADR and HL60/RX cells. Correspondingly, a decrease in expression of pAKT and nuclear p65 was seen in cells treated with LDE225. Because the PI3K/AKT/NF-kB pathway is known to contribute to radiation resistance [26-28], these results support that the Hh pathway inhibitor sensitizes cells exposed to radiation by decreasing the expression of the PI3K/AKT/NF-kB pathway.

Because γ-H2AX foci were increased in the combined treatment group, we next evaluated Rad51 protein expression by western blotting, which is a critical player in homologous recombination (HR) repair. As shown in Figure 3F and 3G, the expression of Rad51 was significantly increased after irradiation of both HL60/ADR and HL60/RX cells as compared with the control. These findings revealed that HL60/ADR and HL60/RX cells exhibit a higher DNA repair capacity that mediates their resistance to radiation. However, both HL60/ADR and HL60/RX cell lines treated with both LDE225 and radiation had no increase in the level of Rad51. These results demonstrate that inhibition of the Hh pathway reduces the capacity for DNA HR repair.

BAK protein has a key role in mitochondrial apoptosis. Consistent with the results of apoptosis analyzed by flow cytometry, combined radiation and LDE225 treatment increased the expression levels of BAK in both cell lines (Figure 3H–3I).

### LDE225 significantly enhances sensitivity to radiation in vivo

**Figure 4:**
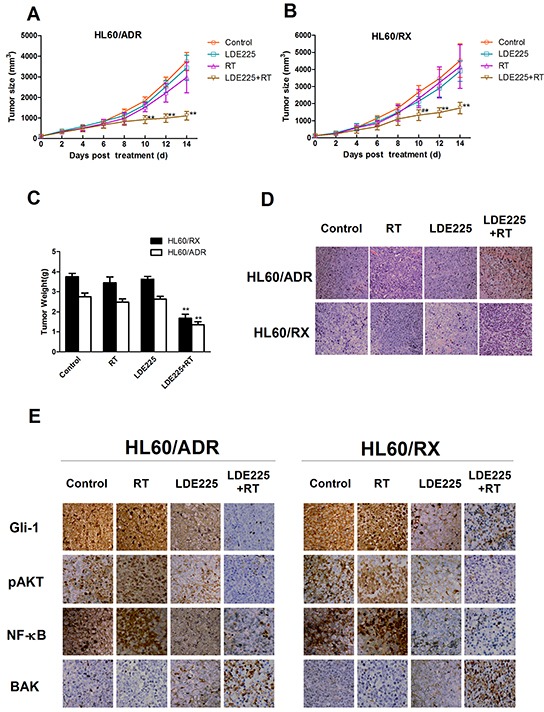
Synergistic effects of LDE225 and radiation on HL60/ADR and HL60/RX cells in vivo Tumors consisting of HL60/ADR and HL60/RX cells were treated in four ways: vehicle alone, radiation alone, LDE225 alone, and combination therapy (LDE225+radiation). Combination therapy significant delays tumor growth both in HL60/ADR **A.** and HL60/RX **B. C.** Nude mice were sacrificed at the 14th day after treatment. The y axis shows the average tumor weight at endpoint. **D.** Histological change of the tumor tissue by HE staining (×400). **E.** Expression of Gli-1, p-AKT, NF-kB, and BAK proteins in each group by IHC after treatments for 14 days (400×). **, p<0.001 vs vehicle-treated control group, radiation group, or LDE225 group; *, p<0.01 vs LDE225 group; #, p<0.001 vs vehicle-treated control group, or radiation group. Abbreviation: RT, radiation.

To validate the radiosensitization of LDE225 in HL60/ADR and HL60/RX cells, we inoculated the two radioresistant leukemia cell lines into the right posterior flanks of nude mice. Tumor-bearing mice were treated as four groups: the vehicle alone as the control, LDE225 alone, radiation alone, and combined LDE225 plus radiation treatment. As shown in Figure 4A and 4B, inoculated tumors grew rapidly in the control, LDE225-alone, and radiation-alone groups, although monotherapy was able to retard tumor growth slightly. However, combined radiation and LDE225 treatment resulted in a significant reduction in the growth of HL60/ADR and HL60/RX xenografts. Consistent with the results of tumor growth, the tumor weight of the combined treatment group was much lower than in other groups (Figure 4C).

To further investigate the effect of LDE225 on enhancing radiosensitivity, histopathological examination and immunohistochemistry (IHC) were performed in all groups. Histopathological examination showed that tumors from the combined treatment group had more multifocal areas of necrosis than other groups (Figure 4D). Protein expression of Gli-1, pAKT, NF-kB, and BAK was assessed by IHC (Figure 4E). Consistent with the in vitro results, both HL60/RX and HL60/ADR tumor cells exhibited high expression of Gli-1 protein, which was reduced after treatment with LDE225. Additionally, the expression of pAKT and nuclear p65 was reduced after LDE225 treatment, which is similar to the data obtained in vitro. Finally, combined radiation and LDE225 treatment increased BAK expression compared with other treatments, and there were no differences among control, LDE225, and radiation groups. These experiments revealed that the protocol of pretreatment with LDE225 followed by irradiation could efficiently inhibit HL60/ADR and HL60/RX tumor growth in vivo, and the mechanism by which LDE225 sensitizes cells to radiation may be mediated through inhibition of the Gli-1/pAKT/NF-kB pathway.

## DISCUSSION

To investigate the effects of Hh signaling on the response to radiation in leukemia cells, we first established radiation-resistant HL60 cells. We found that HL60/RX cells showed increased expression of SMO and Gli-1 compared with the parent line, and resembled HL-60/ADR cells that also express relatively high levels of SMO and Gli-1. When the three cell lines were treated with radiation, HL-60/RX and HL-60/ADR cells exhibited higher resistance than HL-60 cells. Additionally, suppression of Hh signaling using LDE225 significantly enhanced the radiation sensitivity of HL60/RX and HL60/ADR cells both in vitro and in vivo.

The different responses to radiation of cancer cells is largely determined by the level of apoptosis and their DNA repair capacity. The major mechanism of cell killing by radiation is DNA damage. The DNA damage response pathway triggers gene transcription to repair the damage, and unrepaired DNA damage leads to apoptosis. Many previous reports have demonstrated an increase in the capacity to repair DSBs, the main type of DNA damage induced by ionizing radiation [29, 30], as well as reduced apoptosis in radiation-resistant sublines compared with the radiosensitive parent line [31, 32]. Because Hh signaling regulates many genes involved in DNA repair processes, targeting Hh signaling has the potential to increase sensitivity to radiotherapy. For example, Tsai et al. [33] demonstrated that combining cyclopamine, a Hh inhibitor, with radiotherapy enhances radiosensitivity in a hepatocellular carcinoma cell line. In the present study, compared with the parental cell, the radiation-resistant cell line HL60/RX showed a high survival rate after irradiation and overexpression of Hh signaling molecules including SMO and Gli-1. Interestingly, similar to HL60/RX cells, HL60/ADR cells, a drug-resistant leukemia cell line, also showed significant radiation resistance and high levels of SMO and Gli-1. Moreover, both the number of γ-H2AX foci, a biomarker to measure DNA DSBs, and the apoptosis rate were higher in HL60 cells than in HL60/RX and HL60/ADR cells after irradiation. In contrast, we found that treating the two radiation-resistant cell lines with the combination of an Hh inhibitor and radiation significantly increased radiation-induced apoptosis and γ-H2AX foci compared with the control and treatment with single agents. Furthermore, in both HL60/RX and HL60/ADR-xenografted mouse models, the combination of LDE225 and radiation exerted a significant antitumor effect compared with the control and mice treated with single agents. These results strongly indicate that both HL60/RX and HL60/ADR cells are more resistant to radiation than HL60 cells, and overexpression of the Hh signaling pathway may contribute to radioresistance.

Non-homologous end joining and HR are two important pathways for the repair of DSBs [34]. A previous study showed that Rad51 is a HR-specific protein, and high levels of Rad51 enhance resistance to DNA damage [35]. In addition, Rad51-mediated HR is necessary for cell survival in the radioresistance of HL60 cells [36], and downregulation of Rad51 expression may overcome radiation resistance. Here, we also found that both the drug-resistant subline HL60/ADR and radioresistant subline HL60/RX overexpressed the DNA repair pathway protein Rad51 after irradiation, suggesting that the increased ability to repair DNA lesions is a characteristic of radiation resistance in leukemia cells. Subsequently, expression of the apoptotic protein BAK was significantly higher in the combined LDE225 and radiation treatment group than in other groups. Therefore, these results suggest that the increased radioresistance of HL60/RX and HL60/ADR cells is linked to an enhanced ability for DNA repair, and inhibition of the Hh signaling pathway greatly reduces the DNA repair ability and causes a higher rate of apoptosis.

The Hh pathway is a key mediator of many fundamental processes in epithelial repair and regeneration after injury [37]. Hh ligand binds to the receptor Patch, which releases SMO, and then SMO promotes expression of Gli-1 as a transcriptional factor to activate Hh signaling [38, 39]. Furthermore, Hh as a target to improve radiation responses has been reported in recent studies [14, 40]. Crosstalk between Hh and other signaling pathways may partly explain the mechanism of radiosensitivity enhancement. One possible pathway is PI3K/AKT that is implicated in radioresistance [26, 27]. Although the detailed mechanisms of Hh signaling effects on the PI3K/AKT pathway are still unclear, several studies have shown that the activity of AKT can be reduced by Hh inhibitors [41, 42]. Fu et al. [43] reported that Hh protein promotes cell proliferation, migration, and vascular endothelial growth factor production through the PI3K/AKT pathway. Another study has demonstrated that PI3K/AKT signaling is essential for the Hh pathway [44]. Furthermore, a strong biological link between NF-kB and PI3K/AKT pathways in anti-apoptosis has been observed in HL60 cells [45], and deregulated PI3K/AKT and NF-kB activities are the central mediators of cancer cell responsiveness to radiation [28]. Consistent with previous studies, our data obtained from western blot and IHC analyses showed that the activation of pAKT and NF-kB was reduced by LDE225 treatment in both HL60/ADR and HL60/RX cells. Subsequently, downregulation of PI3K/AKT/NF-kB increased apoptosis induced by irradiation, and decreased the DNA repair ability after treatment with LDE225. Thus, targeting Hh signaling would be a potential treatment option to enhance the effectiveness of radiotherapy in leukemia.

## MATERIALS AND METHODS

### Cell culture and reagents

The human myeloid leukemia cell line HL60 and adriamycin-resistant cells (HL60/ADR) were purchased from the Chinese Academy of Sciences (Shanghai, China). HL60 cells were irradiated 12 times over 3 months to produce the radiation-resistant HL60 (HL60/RX) subline (Table 1). HL60, HL60/ADM, and HL60/RX cells were cultured in Roswell Park Memorial Institute (RPMI)-1640 medium (Hyclone, MA, USA) supplemented with 10% heat-inactivated fetal bovine serum (FBS) (Gibco, NY, USA) and 1% penicillin/streptomycin (Life Technologies, Grand Island, NY, USA) in a humidified atmosphere with 5% CO_2_ at 37°C. LDE225, an inhibitor of the Sonic Hh signaling pathway, was purchased from Selleck BioSciences Corporation (Shanghai, China).

### LDE225 and radiation treatments

For in vitro studies, the cells were treated with DMSO (control) or LDE225 (10 μmol/L) in 100-mm culture dishes, and irradiation was performed at room temperature with a 6-MV photon beam produced by a Varian 600 medical linear accelerator at a dose rate of 100 cGy min^−1^. For in vivo studies, tumor-bearing mice were irradiated with a single dose of 4.8 Gy at a dose rate of 100 cGy min^−1^, and LDE225 was orally administered daily at a dose of 80 mg/kg for 10 consecutive days.

### γ-H2AX measurement by immunofluorescence

Cells were collected at 24 h after irradiation and fixed with 4% paraformaldehyde. The cells were washed with phosphate buffered saline (PBS) and then permeabilized by treatment with 0.3% Triton X-100 on ice for 15 min. After blocking with a 4% FBS solution, the cells were incubated with an anti-γ-H2AX antibody (Rabbit, Cell Signaling Technology, MA, USA) at 4°C overnight. After washing, the cells were treated with an anti-rabbit secondary antibody (Dylight 488-conjugated goat anti-rabbit Ig G (H+L), Liankebio, Hangzhou, China) in PBS containg bovine serum albumin (BSA) for 30 min, and then washed twice with PBS in the dark. The cells were incubated with 4′,6-diamidino-2-phenylindoledihydrochloridedihydrate (DAPI) for 20 min, and then coverslips were mounted with anti-fade solution. Nuclear foci were visualized using a confocal fluorescence microscope.

### Clonogenic assay

Cells were seeded into culture plates in RPMI-1640 containing 1% methylcellulose, 30% FBS, and 1% penicillin/streptomycin. After irradiation, the cells were incubated in triplicate at 37°C in a humidified atmosphere with 5% CO_2_ for 10 days. Colonies containing more than 50 cells were counted as one colony, and the colony number was scored under an inverted microscope. The survival curve was then constructed using the Multitarget click model by GraphPad Prism 5.0, and Dq and D0 were calculated. The SER was calculated as the D0 value of radiation plus vehicle treatment divided by the D0 value of radiation plus LDE225 treatment.

### Apoptosis analysis

Apoptosis was measured with a Dead Cell Apoptosis Kit (Invitrogen, UK) using Alexa Fluor 488-conjugated annexin V and propidium iodide. In brief, approximately 1×10^6^ cells were plated in 100-mm dishes. After treatment as described above, the cells were harvested at 48 h post-irradiation (4.8 Gy), washed with PBS, stained according to the manufacturer's protocol, and analyzed by flow cytometry.

### Western blot analysis

Cells received treatments as described above. Treated and untreated cells were harvested at 48 h post-irradiation (4.8 Gy), washed with PBS, and lysed in lysis buffer (Fdbio science, Hangzhou, China) with protease and phosphatase inhibitors (Fdbio science). Nuclear extracts and cytosol extracts were prepared as a published method with a Nuclear and Cytoplasmic Protein Extraction Kit (KeyGEN BioTECH, Jiangsu, China). Protein quantification was performed by a BCA protein assay kit (Fdbio science). Proteins (50 μg) were separated by 10% sodium dodecyl sulfate-polyacrylamide gel electrophoresis, transferred onto a polyvinylidene fluoride membrane, and blocked with a 5% dry milk solution. The membranes were incubated with antibodies in 4% BSA at 4°C overnight and then incubated with a horseradish peroxidase-conjugated anti-rabbit antibody (Fdbio science). The primary antibodies were as follows: rabbit anti-Gli-1 polyclonal antibody (Cell Signaling Technology, MA, USA), rabbit anti-SMO polyclonal antibody (ABclonal Technology, MA, USA), rabbit anti-AKT polyclonal antibody (Cell Signaling Technology), rabbit anti-pAKT polyclonal antibody (Cell Signaling Technology), rabbit anti-H3 polyclonal antibody (Cell Signaling Technology), rabbit anti-NF-kB p65 polyclonal antibody (ABclonal Technology), rabbit anti-Rad51 polyclonal antibody (ABclonal Technology), rabbit anti-BAK polyclonal antibody (ABclonal Technology), and rabbit anti-glyceraldehyde-3-phosphate dehydrogenase antibody (Fdbio science). Immunoblots were developed with a chemiluminescent detection system (Millipore, MA, USA).

### In vivo experiments

Female BALB/c nude mice (6 weeks old) were purchased from the Animal Resources Centre (Guangdong, China). The animals were maintained in caged housing in specific pathogen-free facilities with a 12-h light/dark cycle. Approximately 1×10^7^ HL-60/ADR or HL-60/RX cells were injected subcutaneously into the right posterior flank of each nude mouse. Mice bearing HL-60/ADR or HL-60/RX tumors of 75–150 mm^3^ in volume were assigned randomly to four groups (six mice per group): HL-60/ADR cells + vehicle; HL-60/RX cells + vehicle; HL-60/ADR cells + RT; HL-60/RX cells + RT; HL-60/ADR cells + LDE225; HL-60/RX cells + LDE225; HL-60/ADR cells+LDE225+RT; HL-60/RX cells+LDE225+RT. Mice received the vehicle or LDE225 (80 mg/kg/d) by gavage once daily. After 48 h, radiation and combined treatment groups underwent irradiation (4.8 Gy) as described above. Tumor sizes were calculated every 2 days as: V=0.5×a×b^2^, where a is the longest diameter and b is the shortest diameter of the tumor mass determined using calipers. At 2 weeks, the mice were sacrificed and the tumors were fixed in 10% neutralized formalin overnight after weighing.

### Histopathological examination and IHC staining

The tumor tissues were dehydrated, fixed in paraffin, and cut into 4-μm sections for histopathological examination and IHC. The sections were stained for Gli-1, NF-kB, p-AKT, and BAK protein expression using standard techniques. Hematoxylin and eosin staining was also performed and observed by light microscopy.

### Statistical analysis

Results are presented as the mean±standard deviation. Statistical analysis was performed using the Student's t-test and one-way analysis of variance followed by the Bonferroni test when equal variances were assumed. Dunnett's T3 test was used when equal variances were not assumed. A P-value of less than 0.05 was considered statistically significant.

## CONCLUSIONS

We found that the Hh pathway plays an important role in radiation resistance in both radio- and drug-resistant leukemia cells. Inhibition of the Hh pathway using LDE225 significantly sensitizes HL60/ADR and HL60/RX cells to radiation. Furthermore, our data supported that the effect of LDE225 for radiosensitization may be mediated through downregulation of the Gli-1/PI3K/AKT/NF-kb pathway.
